# Medication adherence and outcomes after paediatric kidney transplantation: results from a telemedicine-based, multimodal aftercare approach

**DOI:** 10.3389/fneph.2025.1569116

**Published:** 2025-06-12

**Authors:** Sinem Karaterzi, Jenny Prüfe, Julia Katharina Wolff, Nele Kirsten Kanzelmeyer, Thurid Ahlenstiel-Grunow, Raoul Gertges, Andrea Dehn-Hindenberg, Mariel Nöhre, Martina De Zwaan, Uwe Tegtbur, Mario Schiffer, Lars Pape

**Affiliations:** ^1^ Department of Pediatrics II, University Hospital of Essen, Essen, Germany; ^2^ IGES Institute, Berlin, Germany; ^3^ Institute for Community Medicine, Department of Prevention Research and Social Medicine, University Medicine, Greifswald, Germany; ^4^ Department of Pediatric Nephrology, Hannover Medical School, Hannover, Germany; ^5^ Department of Psychosomatic Medicine and Psychotherapy, Hannover Medical School, Hannover, Germany; ^6^ Department of Sports Medicine, Hannover Medical School, Hannover, Germany; ^7^ Department of Nephrology, University of Erlangen, Erlangen, Germany

**Keywords:** kidney transplantation, case-management, adherence, graft survival, telemedicine, adolescent, young adult, mental health

## Abstract

**Background:**

Adolescents and young adults demonstrate the poorest long-term graft survival post-kidney transplantation (KTx) due to a multifactorial aetiology. KTx360° is a multicentre, multimodal, telemedicine-based follow-up care model designed to improve transplant survival in adult and paediatric patients.

**Methods:**

The paediatric component of the study was conducted at the Hannover study centres from May 2017 to October 2020 and is registered under the ISRCTN29416382 trial code. The post-transplant care model employed a structured approach, incorporating specialized case management, telemedicine support, psychological assessments and exercise assessments, with targeted interventions. The present study adopted a quasi-experimental, prospective, observational design. The primary endpoint was graft failure, defined as death or the initiation of long-term dialysis. The secondary endpoints were appointment and medication adherence, quality of life, and mental health. In the current study endpoints were analysed in a quasi-experimental, prospective, observational study: All secondary endpoints were analysed longitudinally over study duration in the intervention group using study data. Graft failure was investigated using claims data from participating statutory health insurance providers by a comparison of the eligible-to-treat group (patients transplanted after 2017 (after start of KTx360°) in study centres; ETT) to historical data in study centres (patients transplanted between 2012 and 2017 (before start of KTx360°); historical control group) and two external control groups (controls transplanted after 2017 external control group resp. between 2012–2017 in other KTx centres external historical control group). Descriptive analyses were performed reporting 95% confidence intervals.

**Results:**

We recruited 72 children/adolescents of whom 26 were incident (enrolled within the first year after KTx) and 46 prevalent (enrolled >1 year after KTx) participants. For all participants study data was collected on appointment and medication adherence, quality of life, and mental health. Claims data was available of 22 patients in the ETT, 17 patients in the historical control group, 71 patients in the external control group and 68 patients in the external historical control group (availability of data depends on number of participating insurance companies). In the initial years of the aftercare period, the study data revealed complete adherence behaviour among both prevalent and incident participants. However, a trend towards increasing non-adherence among prevalent participants compared to incident participants was observed. During the observation period in the first year following transplantation, no graft failure was observed in any of the study centre groups: the ETT and historical control group. Low levels of graft failure (3-6%) were observed in the external controls (external control group and external historical control group, other KTx centres). Patients were at increased risk for mental health issues with internalizing symptoms being most prevalent. Parents rated their children’s mental health worse than the patients themselves. While we saw general improvement over the course of the study, changes were not significant. Similar, quality of life was judged worse by-proxy than by patients. Development of quality of life over the course of study was heterogeneous.

**Conclusion:**

The present study observed slight trends of increasing non-adherence among prevalent participants. However, adherence levels remained consistently high across all groups. No graft failures were recorded during the observation period in the study centre Hannover before and after the implementation of KTx360°. Graft survival and adherence were significantly better in the paediatric participants than in adults. The present study suggests that adherence-enhancing and individualized therapies based on telemedicine may potentially be effective over the long term. Assessment of quality of life and mental health revealed an elevated probability of mental health concerns. Evidence from patients and proxies indicated that a combined assessment is an effective method of identifying patients at risk.

## Introduction

For patients with chronic kidney disease stage 5 (CKD5), kidney transplantation (KTx) is the preferred treatment option, offering long-term benefits in terms of survival, reduced morbidity and improved quality of life when compared with dialysis (1, 2). Despite the advent of novel immunodiagnostics and immunosuppression techniques in recent decades, there has been insufficient improvement in long-term graft survival (3).

Acute graft losses are attributable to technical issues and vascular complications, followed by acute rejection and transplant-glomerulonephritis. Consequently, patients with KTx <1 year are closely monitored at the transplant centre. Non-adherence to immunosuppressants has been associated with allograft loss in adolescents (4). The International Paediatric Nephrology Association (IPNA) has described a significant increase of graft failure during the transition to adult care (5). Several meta-analyses have indicated that approximately one-third of graft losses on an annual basis can be attributed to non-adherence in adults (6). In 2005, Hwang et al. published data indicating that adolescent and young adult age groups demonstrate the poorest long-term graft survival (7). Infection, malignancy and metabolic morbidity are the primary causes of paediatric hospitalization and death following transplantation (8, 9). Within the adult population, approximately 8% of patients experience graft loss within the initial three years post-transplantation, with a subsequent steady increase in the years that follow (10). The development of comprehensive strategies, incorporating patient education, regular monitoring, and psychological support, may enhance adherence and consequently improve long-term outcomes (11).

KTx360° has established a structured post-transplant care programme integrating healthcare providers from various sectors of the German health system, including hospital transplant centres and relevant health professionals such as transplant nurses, psychologists and sports medicine specialists. Prior studies suggested that text reminders, problem-solving may each have benefits in transplanted patients (12–14).

The present study aims to develop new strategies to identify and manage modifiable risk factors in KTx recipients, with a particular focus on non-adherence. Despite the advancements in digitalization, post-transplant care in Germany remains largely un-digitalized, which poses an additional barrier to the quality of care. The KTx360° programme was a multicentre, multisectoral, multimodal, telemedicine-based follow-up care model developed to improve transplant survival and consequently reduce healthcare costs by decreasing the need for hospital treatment after KTx in adults and children (11).

## Methods

### Study design

The study participants of the paediatric part of the study were recruited between May 2017 and November 2019 within the structured post-transplant care programme KTx360° under the trial registration ISRCTN29416382. 74 children and adolescents were asked to participate and 72 decided positively. Inclusion criteria were having a KTx or follow-up visit at one of the study centres and having a statutory health insurance. All 36 statutory health insurances (SHIs) of the enrolled patients participated in and endorsed the programme; however, only 27 (covering > 60% of the German population and data of all German transplant centres) submitted claims data for the comparative analyses. There is no bias due to missing data of nine health insurances as problems with data submission resulted only from technical problems and are unrelated to any patient characteristics. The paediatric element of the project was conducted at Hannover Medical School in Lower Saxony, Germany. A detailed description of the methodology is available in the published study protocol (11).

**Figure 1:**
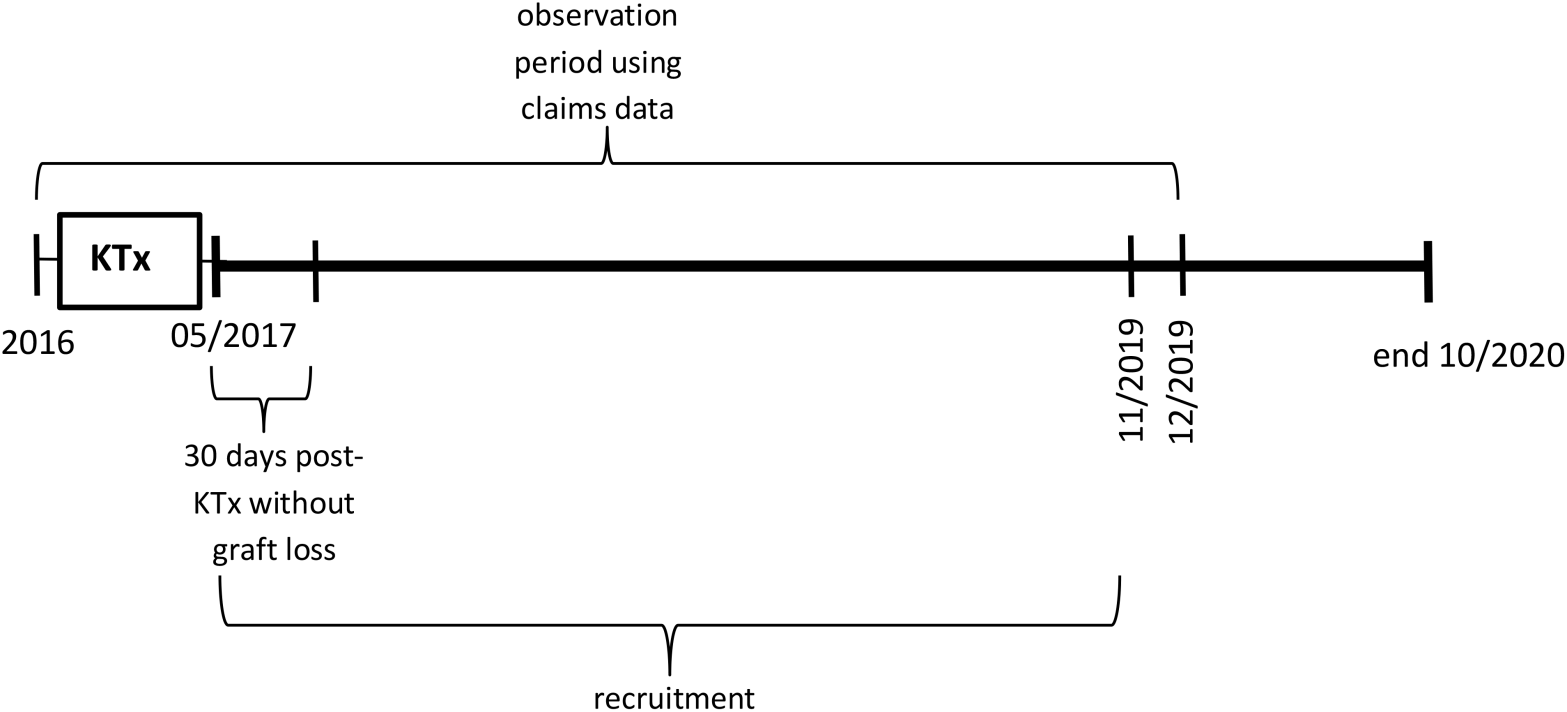
Observation periods using claims data relative to the timepoint of kidney transplantation (KTx) for incident participants and controls.

**Figure 2:**
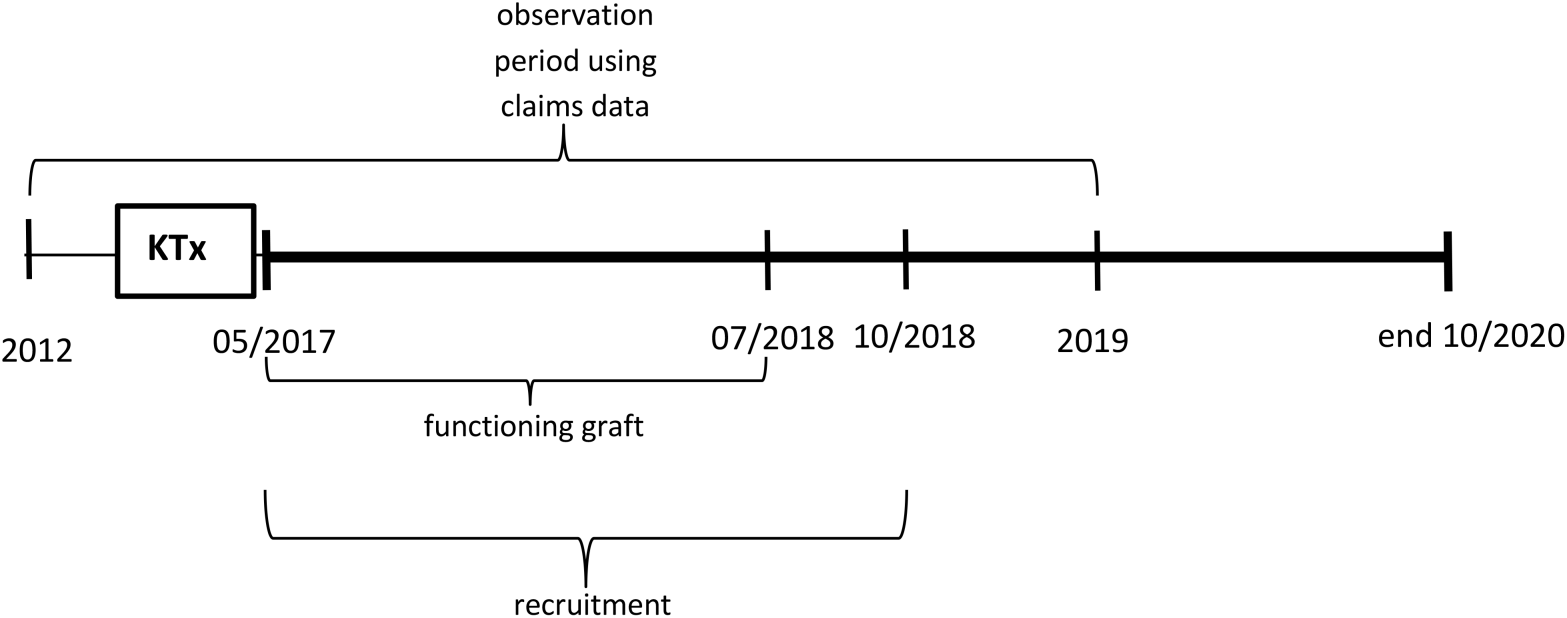
Observation periods using claims data relative to the timepoint of kidney transplantation (KTx) for prevalent participants and controls.

Two cohorts of patients were included in KTx360°: incident and prevalent patients. Incident patients had their KTx during the recruitment period of the study at one of the two children transplant centres. Prevalent patients had their KTx prior between 2010 and 2016 and participated in follow-up care visits at the transplant centres. The control arm consists of claims data from KTx patients who did not participate in the programme because they were treated before programme start in Hannover or in parallel in other German centres (**Figures 1**, **2**).

### Ethics statement

Ethics approval was obtained from Hannover Medical School and all guardians of the paediatric participants gave written informed consent.

### Interventions

The KTx360° programme focused on the management of post-transplant patients through the implementation of eHealth components and a range of integrated therapeutic interventions. These interventions are comprehensively described in the study protocol (11). The project incorporated case management and an internet-based case file to document for each team member (‘CasePlus^®^’, Symeda GmbH, Germany). In addition, the project entailed the conduction of periodic psychosocial and cardiovascular risk assessments. Training goals were established for each patient on an individual basis. Each patient underwent evaluations by a mental health specialist, including analyses of non-adherence to immunosuppressants and identification of individual barriers to adherence optimization using the Basel Assessment of Adherence with Immunosuppressive Medication Scale (BAASIS^©^) (15, 16).

Routine mental health screenings were completed upon inclusion and followed by annual assessments, usually alongside the yearly transplant follow-up. Initial assessment consisted of a screening using CBCL followed by a clinical interview of up to 90 minutes. In case of mental health issues, families initially received a 1-hour counselling session which aimed at both parents and child. Here, the problem was contextualized and goals were identified. Follow-up sessions were scheduled alongside the medical appointments (most commonly once a month). Focus was on education and coping strategies. Number of appointments depended on the type and gravity of problems. In case of mental illness requiring psychotherapy, referrals were made.

Depending on the results of the adherence assessments, the psychosocial team recommended educational groups or individual adherence coaching. A maximum of eight sessions per year could be provided by a member of the mental health team. The provision of adherence coaching and individualized sports therapy was based on the results of the assessments. The provision of coaching was facilitated through either video conferencing or in-person consultations. The case managers, who were experienced transplant nurses, were responsible for coordinating individual post-transplant care and offering continuous support to patients. All study participants were invited to participate in all modules of the programme.

### Sample and data

The sample of study participants comprised 46 prevalent children/adolescents and 26 incident children/adolescents. Incident patients were recruited from May 2017 until October 2018 and prevalent patients between May 2017 and November 2019. Duration of study participations was on average 26.33 months in incident (SD = 8.34, Med = 28.32, Min = 10.53, Max = 38.53) and 32.56 in prevalent patients (SD = 8.30, Med = 33.58, Min = 6.77, Max = 41.07).

*Study data.* For all participants study data are available for medication adherence (BAASIS interview, self-report or proxy-report) and intra-patient variability of immunosuppressants (IPV)) (17). For BAASIS, medication adherence is defined as reporting adherence to all five questions of the interview. That is, non-adherence indicates reported non-adherence to at least one question of the BAASIS. IPV is a surrogate associated with reduced adherence to immunosuppressants and an increased risk of poor outcomes following kidney transplantation (18). Additionally, mental health status was assessed using the age-appropriate by-proxy reports of Achenbach Child Behaviour Checklist (CBCL) as well as the teenager’s version (YSR) in participants aged 11 years and older. Quality of life was assessed using the PedsQL transplant model in the age-relevant versions for self- and by-proxy-reports. Participants were observed until allograft failure or their last patient follow-up date as indicated in the registry.

*Claims data.* Claims data were available from 27 health insurance companies covering data from study centres and other transplant centres in Germany comprising data of transplantation between 2012 and 2019. For analyses four groups were defined: (1) The eligible-to-treat group (ETT) with all patients transplanted after 2017 in study centres (covering the time after implementation of KTx360°). (2) A historical control group with transplantation before 2017 at study centres. (3) An external control group with transplantation after 2017 at other transplantation centres. (4) An external historical control group with transplantation before 2017 at other transplantation centres. For analyses the ETT was compared to all three control groups controlling for change over time in the same transplantation centre (historical control group vs. ETT) and for change over time and study participation in other transplantation centres (external historical control group vs. ETT and external control group vs. ETT). Graft failure in year one and two after KTx was defined as death or initiation of long-term dialysis (a minimum of 5 dialysis sessions within the corresponding observation period).

### Statistical analysis

The was analysed using descriptive statistics and the absolute number of missing values was indicated for each variable. Data of psychometric assessments were calculated based on the relevant test manuals. CBCL-/YSR-Data are reported as T-scores and percentiles. Between group differences were calculated using chi-square tests, Fisher exact tests, t-tests, and Mann-Whitney-U-tests as appropriate.

### The role of the funding source

The Innovation Fund of the Joint Federal Committee of the Federal Republic of Germany provided financial support for the study (grant number 01NVF16009). The Committee played no part in the design and execution of the study, the analysis and interpretation of the data, the preparation, review, or approval of the manuscript, or the decision to submit the manuscript for publication. All authors had full access to all the data from the study and accept responsibility for the decision to submit the manuscript for publication.

## Results

### Participants’ characteristics

The characteristics of the participants are documented in **Tables 1**, **2**. In total, 72 children and adolescents were included in this project. Of these, 46 were identified to belong the prevalent group and 26 to the incident group. The mean age at the time of enrolment was 11.23 years (SD 4.77); approximately 55% of the participants were male.

**Table 1:** Characteristics of prevalent and incident participants at enrolment into KTx360°.

Prevalent and Incident participants			
	Incident (N = 26)	Prevalent (N = 46)	Total (N = 72)
Age, years			
Median	9.5	12.3	11.29
Sex, n (%)			
Male	14 (53.8%)	25 (54.3%)	39 (54.2%)
Female	12 (46.2%)	21 (45.7%)	33 (45.8%)
Transplantation center, n (%)			
Hannover Medical School	25 (96.2%)	46 (100%)	71 (98.6%)
Hannoversch Muenden in Lower Saxony	–	–	–
Erlangen	1 (3.8%)	–	1 (1.4%)
Donation type, n (%)			
Missing	–	1 (2,2%)	1 (1.4%)
Living	6 (23.1%)	15 (32.6%)	21 (29.2%)
Deceased	20 (76.9%)	30 (65.2%)	50 (69.4%)
Previous KTx, n (%)			
Yes	2 (7.7%)	8 (17.4%)	10 (13.9%)
Diabetes mellitus, n (%)			
Yes	–	1 (2.2%)	1 (1.4%)
Hypertension, n (%)			
Yes	12 (46.1%)	32 (69.6%)	44 (61.1%)

**Table 2:** Sample characteristics at time of KTx.

Prevalent and Incident participants			
	Prevalent (N = 46)	Incident (N = 26)	Total (N = 72)
Duration of kidney insufficiency, months			
Median	35.6	28.2	41.5
Duration of dialysis, months			
Median	11.8	15.6	13.3
Type of dialysis, n (%)			
Hemodialysis	9 (19.6%)	8 (30.8%)	17 (23.6%)
Peritoneal dialysis	21 (45.6%)	10 (38.4%)	31 (43.1%)
Preemptive transplantation	16 (34.8%)	8 (30.8%)	24 (33.3%)
Dialysis after KTx, n (%)			
Yes	3 (6.5%)	2 (7.7%)	5 (6.9%)
HLA mismatches (A. B. DR)			
Missing	1 (2,2%)	–	1 (1,4%)
Median	3.0	3.6	3.2
Induction extracorporeal procedure, n (%)			
Immunoadsorption	2 (4.4%)	1 (3.9%)	3 (4.2%)
Plasmapheresis	–	1 (3.9%)	1 (1.4%)
None	44 (95.6%)	24 (92.2%)	68 (94.4%)
Antibody induction, n (%)			
Missing	6 (13%)	6 (23,1%)	12 (16,7%)
ATG	–	2 (7.7%)	2 (2.8%)
ATG + Basiliximab	–	–	–
Basiliximab	40 (87%)	18 (69.2%)	58 (80.5%)

Data taken from study data.

KTx, kidney transplantation, (28, 30), HLA, human leukocyte antigen, ATG, Anti-Thymocyte Globulin.

### Appointment adherence (study data)

A total of 100% adherence was observed among transplant recipients (KTx<1/2 year) in regard to their scheduled follow-up appointments. In the fourth year of aftercare, a slight decrease in appointment-adherence was observed compared to the first year (99.4% and 96.23%, respectively) (**Table 3**). Notwithstanding this slight decrease, adherence remained very high.

**Table 3:** Appointment adherence.

Time after Tx	N patients	N scheduled appointments	M	SD	KI 95%	
					LO	HI
1–30 days	8	14	100.00%	–	–*	–*
31–90 days	16	87	100.00%	–	–*	–*
91–180 days	23	133	100.00%	–	–*	–*
181–360 daya	30	262	98.08%	9.9%	94.49%	100.00%
Year 1	30	510	99.44%	2.8%	98.47%	100.00%
Year 2	33	336	96.71%	9.2%	93.01%	99.28%
Year 3	32	235	97.94%	8.97%	94.53%	100.00%
Year 4+	46	910	96.23%	5.85%	94.46%	97.79%
total	71	1994	96.80%	5.99%	95.31%	98.07%

### Medication adherence (study data)

**Figure 3:**
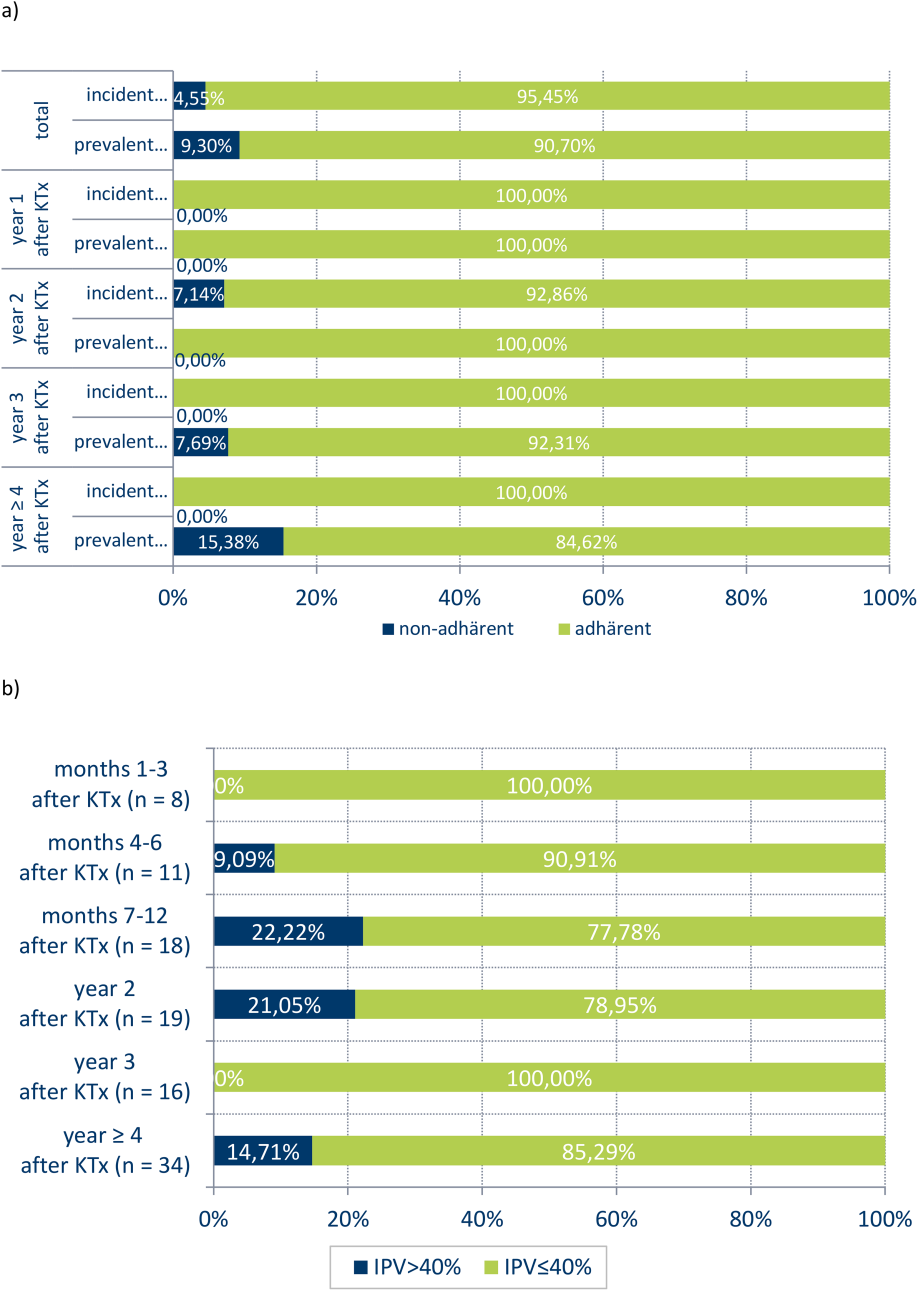
Medication adherence by BAASIS-Scale **(a)** and IPV **(b)**.

The analyses of BAASIS scores on medication adherence revealed that prevalent and incident participants exhibited comparable medication adherence behaviour in the initial year after transplantation. Subsequent years revealed that all groups maintained an adherence rate of at least 85%. However, a total of 4.55% of incident and 9.30% of prevalent participants exhibited self-reported non-adherence (**Figure 3a**).

Additionally, the intra-patient variability of immunosuppressants (IPV) was measured. Data on IPV was available for 39 patients across six time periods. A person was included in the analyses for a specific time period after KTx, if there are at least three measures of immunosuppressant trough levels available in this time period. In the first three months following KTx, there was no documented high IPV (i. e. IPV ≤.40). However, between six months after KTx and up to the second year, we observed a peak in IPV increase, reaching 22.22%. In the fourth year of aftercare, we identified a slight decrease in IPV to 14.7% (**Figure 3b**).

### Graft failure

Graft loss was not observed during year one and two in the ETT and historical control group (both with transplantations in study centres). However low levels of graft failures are documented in the first but not second year after KTx in the two external CGs. Results indicate descriptively for the first year after KTx, that patients in study centres have a slightly lower risk of graft failure than in other KTx centres. However, change in graft failure from before start of KTx360° to after start of KTx360° in study centres is not observable, as no graft failure occurred in both groups (ETT and historical control group) (**Table 4**). Because of the small sample size and because there is no variance on graft failure in the ETT and historical control group, no significance test of the result is included.

**Table 4:** Graft failure.

	Years after Tx							
	1^st^ year				2^nd^ year			
	N	M	95%-KI		N	M	95%-KI	
			LO	HI			LO	HI
Historic control group	17	0.00%	–*	–*	16	0,00%	–*	–*
Eligible-to-Treat-group (ETT)	22	0.00%	–*	–*	7	0.00%	–*	–*
External historic control group	68	2.94%	0.36%	10.22%	84	0.00%	–*	–*
External control group	71	5.63%	1.56%	13.80%	31	0.00%	–*	–*

For more details the characteristics of the study groups based on claims data are shown in **Table 5**.

**Table 5:** Characteristics of study groups in claims data at KTx.

	Historic control group		Eligible-to-treat-group (ETT)		External historic control group		External control group	
	n	%	n	%	n	%	n	%
Female	6	35.29%	10	45.45%	25	36.76%	28	39.44%
Hypertension	1	5.88%	8	36.36%	6	8.82%	3	4.23%
CVD	2	11.76%	0	0.00%	5	7.35%	5	7.04%
Diabetes	2	11.76%	0	0.00%	0	0.00%	0	0.00%
Mental and behavioral disorder	1	5.88%	4	18.18%	6	8.82%	9	12.68%
Pre-transplantation	0	0.00%	2	9.09%	30	44.12%	19	26.76%
Body donation	14	82.35%	18	81.82%	47	69.12%	47	66.20%
**Total**	**17**		**22**		**68**		**71**	

### Mental health and quality of life

As part of the programme patients underwent regular mental health screening and standardized assessment of quality of life. Inclusion into standardized assessment depended on age as well as on cognitive and physical abilities.

Mental-health was screened in 46 patients (11 incident and 35 prevalent) aged 6 years and above using the Child Behaviour Checklist (by-proxy version) at time of study start (T0) and 12 months later (T1). Of these 46 patients, four had only the follow-up assessment as they were too young for mental health screening when included into the study. At time of inclusion, by-proxy reports indicated that 5 (10.87%) patients were at risk for mental health issues by their overall-T-score. In 12 cases (26.07%) patients’ clinical symptoms were reported. Internalizing symptoms were reported twice as often as externalizing symptoms. Regarding the follow up, it should be noted that 10 participants did not complete the mental health assessment. **Table 6** shows the profile of clinical symptoms grouped according to DSM-categories.

**Table 6:** Mental health – by-proxy reports (Timepoint 0 study start, Timepoint 1 + 12 months).

Category	M ± SD	<94. Perc	94–97 perc	>97 perc
Depression Timepoint 0	52.26 ± 8.29	24 (57.14%)	9 (21.43%)	9 (21.43%)
Depression Timepoint 1	57.00 ± 8.32	19 (73.08%)	0 (0.00%)	7 (26.92%)
Anxiety Timepoint 0	57.88 ± 12.00	26 (61.91%)	4 (9.52%)	12 (28.57%)
Anxiety Timepoint 1	57.42 ± 7.63	17 (65.39%)	2 (7.69%)	7 (26.92%)
ADH Timepoint 0	56.79 ± 7.22	29 (69.05%)	3 (7.14%)	7 (16.67%)
ADH Timepoint 1	58.64 ± 9.05	18 (69.23%)	1 (3.85%)	7 (26.92%)
Oppositional behavior Timepoint 0	55.88 ± 7.43	32 (76.19%)	5 (11.91%)	5 (11.91%)
Oppositional behavior Timepoint 1	57.00 ± 8.19	18 (69.23%)	3 (7.14%)	5 (19.23%)
Dissocial behavior Timepoint 0	55.21 ± 7.49	33 (78.57%)	5 (11.91%)	4 (9.52%)
Dissocial behavior Timepoint 1	54.92 ± 5.46	21 (80.77%)	4 (15.39%)	1 (3.85%)

While not statistically significant, paired t-test results of 22 participants who completed CBCL by-proxy reports at time of inclusion and after completion of the programme revealed mild improvements over time across all diagnostic domains, with the exception of oppositional behaviour. Mildest changes were observed in aspects of attention deficit and hyperactivity (mean -0.27, p=0.84); the strongest changes were observed in the overall score of internalizing symptoms (mean 4.36, p=0.05).

At the time of inclusion, incidental patients exhibited a reduced prevalence of mental health challenges in comparison to the prevalent group. Group comparison using the Mann-Whitney U-test revealed significant differences in the domains of ADH (p=0.01), dissocial behaviour (p=0.05), the sum score of internalizing symptoms (p=0.03), and the overall sum score (p=0.01). However, this levelled out and at timepoint 1, no such differences could be observed.

Furthermore, 28 teenagers (12 male, 16 female) completed the self-report forms (YSR). Seven participants (25%) were from the incidental group, and 19 teenagers (67.86%) were assessed at least twice; the remaining nine were not old enough for YSR assessment at the time of inclusion. The results did not correlate with by-proxy reports. Generally, the teenagers regarded themselves as having better mental health compared to their parents’ judgements. Overall, parents assigned their child a lower quality of life rating than the patients themselves At the time of inclusion, 5 (17.86%) teenagers reported being at risk of mental health issues; however, none had sum-scores that indicated the presence of relevant clinical issues.

Paired t-test results of the 19 participants who completed all assessments (by proxy and self-report forms) demonstrated mild improvements over time in internalizing symptoms, while externalizing symptoms slightly worsened. The sum score of internalizing symptoms demonstrated a significant improvement (mean 3.37, p=0.047). The study revealed no statistically significant group differences between incident and prevalent patients or based on sex, as determined by the Mann-Whitney U test.

**Table 7** shows the profile of clinical symptoms according to DSM-categories.

**Table 7:** Mental health - self-report (Timepoint 0 study start, Timepoint 1 + 12 months).

Category	M ± SD	<94. Perc	94–97 perc	>97 perc
Depression Timepoint 0	56.29 ± 7.55	21 (75.00%)	3 (10.71%)	4 (14.29%)
Depression Timepoint 1	54.68 ± 4.89	17 (89.47%)	1 (5.26%)	1 (5.26%)
Anxiety Timepoint 0	55.96 ± 6.47	20 (71.43%)	5 (17.86%)	3 (10.71%)
Anxiety Timepoint 1	54.47 ± 7.12	14 (73.68%)	2 (10.53%)	3 (15.79%)
ADH Timepoint 0	54.75 ± 5.77	21 (75.00%)	5 (17.86%)	2 (7.14%)
ADH Timepoint 1	53.16 ± 4.14	18 (94.74%)	1 (5.26%)	0
Oppositional behavior Timepoint 0	52.25 ± 3.95	26 (92.86%)	1 (3.57%)	1 (3.57%)
Oppositional behavior Timepoint 1	52.84 ± 3.88	16 (84.21%)	1 (5.26%)	2 (10.53%)
Dissocial behavior Timepoint 0	53.46 ± 4.6	24 (85.1%)	3 (10.71%)	1(3.57%)
Dissocial behavior Timepoint 1	52.95 ± 3.88	18 (94.74%)	1 (5.26%)	0

A total of 57 parents and 44 patients contributed to the quality-of-life assessment. Overall, parents assigned their child a lower quality of life rating than the patients themselves. Conversely, children’s self-reports exhibited a favourable trend over the course of the study, albeit not reaching statistical significance. Parents, however, reported a decline in social interaction skills, as indicated by the domains “my transplant and others” and “communication,” along with heightened concerns regarding general worries and treatment-related anxieties (**Table 8**).

**Table 8:** Quality of Life (Timepoint 0 study start, Timepoint 1 + 12 months).

PedsQL scale	mean ± SD by proxy (n=57)	mean ± SD Patient (n=44)	Paired t-test when significant
My medication 1 Timepoint 0	80.00 ± 18.97	85.65 ± 12.36	
My medication 1 Timepoint 1	83.61 ± 16.68	87.17 ± 12.66	
My medication 2 Timepoint 0	77.69 ± 18.24	80.08 ± 17.40	
My medication2 Timepoint 1	77.29 ± 20.36	84.29 ± 13.07	
My transplant and others Timepoint 0	65.96 ± 20.45	69.89 ± 18.10	t(40)=2.62, p=0,012
My transplant and others Timepoint 1	62.29 ± 15.59	77.19 ± 14.12	t(26)=5.02, p=0.00
Pain Timepoint 0	70.98 ± 22.25	74,81 ± 23.81	t(40)=2.78, p=0.00
Pain Timepoint 1	71.75 ± 22.12	82.78 ± 19.69	t(26)=2.77, p=0.01
worries Timepoint 0	62.37 ± 25.35	74.27 ± 18.53	t(40)=3.42, p=0.00
worries Timepoint 1	58.47 ± 23.10	76.79 ± 20.37	t(26)=2.69, p=0.01
Treatment anxiety Timepoint 0	64.25 ± 30.08	70.06 ± 27.16	
Treatment anxiety Timepoint 1	60.93 ± 30.97	77.03 ± 21.67	
How I look Timepoint 0	69.30 ± 26.36	64.92 ± 31.26	
How I look Timepoint 1	61.18 ± 20.08	71.84 ± 23.93	t(25)=2.80, p=0.01
Communication Timepoint 0	63.49 ± 29.40	69.94 ± 25.12	t(40)=2.21, p=0.03
Communication Timepoint 1	58.43 ± 27.10	77.92 ± 19.26	t(26)=3.67, p=0.00

## Discussion

KTx360° is a pioneering project in the field of integrated care models. The post-transplant care programme utilized multimodal and multidisciplinary interventions, with the objective of reducing healthcare expenditures, optimizing post-transplant care and enhancing quality of life.

We recruited 72 children, the majority of whom are from the Hannover Medical School. On a national scale, approximately 100 paediatric kidney transplants are performed annually in Germany (19). Given that the Hannover Medical School is one of the major transplantation centres in the country, the results from this cohort can be considered representative of paediatric kidney transplantation practices in Germany.

Immunosuppressant/medication non-adherence has been demonstrated to result in rejection and early graft failure (20). Cechka et al. identified a poor 5-year graft survival rate among 13- to 21-year-old kidney transplant recipients in the United Network for Organ Sharing analyses (21). Further recent clinical studies have corroborated the elevated risk of graft failure in adolescents (22, 23).

The present study revealed a trend of increasing suboptimal adherence in prevalent participants compared to incident participants. However, no significant differences were observed between the controls and participants in terms of appointment adherence. The scheduled follow-up appointments were fully adhered to within the first year in all groups. These high rates of appointment adherence in the present study’s patients were probably based on the well-organized outpatient unit. Similarly, there is high rate in medication adherence in incident and prevalent patients, with a slightly higher rate of non-adherence in prevalent patients that may be caused by their higher age. High tacrolimus IPV has been associated with poor outcomes and increasing the risk of immune-mediated rejection (16, 24). In the first three months post-KTx, no high tacrolimus IPV was identified; however, a marginal increase in IPV was observed later, suggesting fluctuating IPV, which is comparable to the data from our adult cohort.

The study hypothesized that parents play a crucial role in adherence of younger children, and that they are the key figures in ensuring adherence (25). However, as children enter adolescence, it is hypothesized that parental control likely diminishes, and the adolescents’ self-responsibility increases, at which stage parental influence becomes minimal. This might also be one of the reasons why adherence tends to decrease during adolescence, as described in the literature (26). The data showed a trend of decreasing medication adherence to follow-up appointments over the years. As demonstrated in our previous publication by Pape et al., a significant improvement in graft survival was observed in the adult prevalent study group in comparison to propensity score-matched controls with KTx360° (27). With a graft survival rate of 100%, it was not possible to identify any improvements in our paediatric cohort. No cost savings were achieved through the programme, as the patients already incurred high expenses due to intensive care. Cost savings through the prevention of graft failure could not be assessed, as no graft failures occurred at any point during the study. Long term cost effectiveness according to better graft survival remains speculative. However, the benefits observed in adults undergoing KTx360° suggest the potential for enhanced long-term survival outcomes in adolescents as well.

Mental health screening resulted in three core-findings: 1) paediatric transplant recipients are at greater risk for mental health problems, particularly for internalizing symptoms; 2) by-proxy reports more frequently exceeded the cut-off scores than patient self-reports; and 3) while internalizing symptoms appear to decrease over time, externalizing symptoms may aggravate.

The present findings are consistent with the conclusions of earlier studies, which reported an increase in mental health symptoms – particularly of depression and anxiety - in children with CKD5 (28–30).

On average, parents tended to report higher levels of symptomatology than their children, a phenomenon that has been observed across various medical and normative populations (31, 32). Conversely, the findings of this study demonstrated that parents perceived their children to be experiencing considerably more symptoms of anxiety and depression than the children themselves and exceeded the results as anticipated based on general population norms. Upon the consideration of the available data, it appears unfeasible to determine whether parents exhibit greater objectivity in their reporting of their children’s mental health symptoms, or whether they are influenced by their own fears and stresses, leading to the projection of these sentiments onto their offspring. Interestingly, patients were not flagged as having mental health concerns by the time of inclusion and thus were untreated until the time of detection. Also, we can only speculate on the increase of externalizing symptoms, particularly those perceived as hyperactivity and attention problems. Transplantation instigates a plethora of changes, the most significant of which pertains to alterations in medication and an enhancement in physical fitness. Patients often report feelings of increased strength, health, energy and decreased fatigue. However, this newfound energy can be perceived by others as hyperactivity. In addition, research literature reports deficits in executive function in children following renal transplantation, with the mechanisms of this phenomenon remaining unknown (33, 34). It is possible that the combination of enhanced physical well-being and attentional impairments, such as deficits in control and inhibition, may lead to parents perceiving an increase in externalizing symptoms.

While patients reported an enhancement in quality of life throughout the study period, parental reports exhibited substantial discrepancies. The phenomenon of discrepancies in inter-rater agreement between child and proxy reporting of quality of life is well-documented (35). Although present data do not provide an explanation, it is imperative to consider the potential for by-proxy bias, whereby parents may possess heightened awareness of future prospects, including the possibility of disease recurrence or transplant failure, which could influence their judgment.

### Limitations

Due to the small number of paediatric patients, drawing definitive conclusions is limited. Additionally, the relatively short follow-up period may represent a potential limitation that could influence the study’s conclusions. To achieve significant results, a larger number of participants would have been required, but such a number is extremely difficult to obtain in paediatrics. The mean age of the study participants was 11 years, which may be indicative of the absence of graft failure across all groups during the study period. The risk of graft failure during late adolescence and early adulthood was not fully captured within the study period. It is important to emphasize that the combined approach utilized in this project may result in more sustainable outcomes for incident participants in the long term; however, this could not be demonstrated in the present study due to its relatively short observation period. The utilization of claims data was mandated by the funding agency, thus precluding the implementation of a randomized controlled trial (RCT), which is widely regarded as the gold standard for therapeutic clinical trials. However, claims data offer the advantage of reflecting trial performance in real-world settings. Nevertheless, potentially significant covariates, such as comorbidities or immunosuppression regimens, are not included in claims data. The principal limitation of the quasi-experimental design is that it does not permit causal attributions of effects, particularly with regard to longitudinal changes without control conditions.

In conclusion, our findings suggest a good appointment and medication adherence within the first years for paediatric patients after kidney transplantation. Our paediatric cohort effectively displayed better adherence-rates, patient, and allograft survival as compared to the adult cohort. Given the superior outcome, it is self-evident, that the KTX360° programme can hardly further improve adherence. Nevertheless, it can be hypothesized that the aftercare programme will eventually contribute to the maintenance of optimal adherence over an extended period. As post-transplant care is relatively similar in all German paediatric centres, we hypothesize that our programme can be generalized. This is supported by the fact that the adult part of KTx360° was successfully implemented in three different centres.

## Data Availability

The raw data supporting the conclusions of this article will be made available by the authors, without undue reservation.

## References

[1] Butani L Perez RV. Effect of pretransplant dialysis modality and duration on long-term outcomes of children receiving renal transplants. Transplantation. (2011) 91:447–51. doi: 10.1097/TP.0b013e318204860b 21131898

[2] Amaral S Sayed BA Kutner N Patzer RE. Preemptive kidney transplantation is associated with survival benefits among pediatric patients with end-stage renal disease. Kidney Int. (2016) 90:1100–8. doi: 10.1016/j.kint.2016.07.028 PMC5072842 27653837

[3] Pascual M Theruvath T Kawai T Tolkoff-Rubin N Cosimi AB. Strategies to improve long-term outcomes after renal transplantation. N Engl J Med. (2002) 346:580–90. doi: 10.1056/NEJMra011295 11856798

[4] Holmberg C Jalanko H. Long-term effects of pediatric kidney transplantation. Nat Rev Nephrol. (2016) 12:301–11. doi: 10.1038/nrneph.2015.197 26656457

[5] Watson AR Harden P Ferris M Kerr PG Mahan J Ramzy M. Transition from pediatric to adult renal services: a consensus statement by the International Society of Nephrology (ISN) and the International Pediatric Nephrology Association (IPNA). Pediatr Nephrol. (2011) 26:1753–7. doi: 10.1007/s00467-011-1981-z 21842231

[6] Pabst S Bertram A Zimmermann T Schiffer M de Zwaan M. Physician reported adherence to immunosuppressants in renal transplant patients: Prevalence, agreement, and correlates. J Psychosom Res. (2015) 79:364–71. doi: 10.1016/j.jpsychores.2015.09.001 26526310

[7] Hwang AH Cho YW Cicciarelli J Mentser M Iwaki Y Hardy BE. Risk factors for short- and long-term survival of primary cadaveric renal allografts in pediatric recipients: a UNOS analysis. Transplantation. (2005) 80:466–70. doi: 10.1097/01.tp.0000168090.19875.b0 16123719

[8] Smith JM Stablein DM Munoz R Hebert D McDonald RA. Contributions of the transplant registry: the 2006 annual report of the North American pediatric renal trials and collaborative studies (NAPRTCS). Pediatr Transplant. (2007) 11:366–73. doi: 10.1111/j.1399-3046.2007.00704.x 17493215

[9] Kirk AD Mannon RB Swanson SJ Hale DA. Strategies for minimizing immunosuppression in kidney transplantation. Transpl Int. (2005) 18:2–14. doi: 10.1111/j.1432-2277.2004.00019.x 15612977

[10] Kasiske BL. Cardiovascular disease after renal transplantation. Semin Nephrol. (2000) 20:176–87. 10746859

[11] Pape L de Zwaan M Tegtbur U Feldhaus F Wolff JK Schiffer L. The KTx360 degrees -study: a multicenter, multisectoral, multimodal, telemedicine-based follow-up care model to improve care and reduce health-care costs after kidney transplantation in children and adults. BMC Health Serv Res. (2017) 17:587. doi: 10.1186/s12913-017-2545-0 28830408 PMC5568357

[12] Miloh T Annunziato R Arnon R Warshaw J Parkar S Suchy FJ. Improved adherence and outcomes for pediatric liver transplant recipients by using text messaging. Pediatrics. (2009) 124:e844–50. doi: 10.1542/peds.2009-0415 19822583

[13] Reese PP Bloom RD Trofe-Clark J Mussell A Leidy D Levsky S. Automated reminders and physician notification to promote immunosuppression adherence among kidney transplant recipients: A randomized trial. Am J Kidney Dis. (2017) 69:400–9. doi: 10.1053/j.ajkd.2016.10.017 27940063

[14] Demonceau J Ruppar T Kristanto P Hughes DA Fargher E Kardas P. Identification and assessment of adherence-enhancing interventions in studies assessing medication adherence through electronically compiled drug dosing histories: a systematic literature review and meta-analysis. Drugs. (2013) 73:545–62. doi: 10.1007/s40265-013-0041-3 PMC3647098 23588595

[15] Denhaerynck K Dobbels F Košťálová B De Geest S BAASIS Consortium. Psychometric properties of the BAASIS: A meta-analysis of individual participant data. Transplantation. (2023) 107:1795–809. doi: 10.1097/TP.0000000000004574 PMC10358438 36949037

[16] Vrijens B De Geest S Hughes DA Przemyslaw K Demonceau J Ruppar T. A new taxonomy for describing and defining adherence to medications. Br J Clin Pharmacol. (2012) 73:691–705. doi: 10.1111/j.1365-2125.2012.04167.x 22486599 PMC3403197

[17] Denhaerynck K Schäfer-Keller P Young J Steiger J Bock A De Geest S. Examining assumptions regarding valid electronic monitoring of medication therapy: development of a validation framework and its application on a European sample of kidney transplant patients. BMC Med Res Methodol. (2008) 8:5. doi: 10.1186/1471-2288-8-5 18284675 PMC2275282

[18] Morais MC Soares ME Costa G Guerra L Vaz N Codes L Bittencourt PL. Impact of tacrolimus intra-patient variability in adverse outcomes after organ transplantation. World J Transplant. (2023) 13:254–63. doi: 10.5500/wjt.v13.i5.254 PMC10514747 37746041

[19] dso, G.O.P.O. Jahresbericht organspende und transplantation in Deutschland 2023 (2024). Available online at: https://dso.de/SiteCollectionDocuments/DSO-Jahresbericht%202023.pdfsearch=nierentransplantation%20kinder (Accessed January 21, 2025).

[20] Denhaerynck K Burkhalter F Schäfer-Keller P Steiger J Bock A De Geest S. Clinical consequences of non adherence to immunosuppressive medication in kidney transplant patients. Transpl Int. (2009) 22:441–6. doi: 10.1111/j.1432-2277.2008.00820.x 19144090

[21] Cecka JM Gjertson DW Terasaki PI. Pediatric renal transplantation: a review of the UNOS data. United Network for Organ Sharing. Pediatr Transplant. (1997) 1:55–64. 10084788

[22] Smith JM McDonald RA North American Pediatric Renal Transplant Cooperative Study. Renal transplant outcomes in adolescents: a report of the North American Pediatric Renal Transplant Cooperative Study. Pediatr Transplant. (2002) 6:493–9. doi: 10.1034/j.1399-3046.2002.02042.x 12453202

[23] Foster BJ Dahhou M Zhang X Platt RW Samuel SM Hanley JA. Association between age and graft failure rates in young kidney transplant recipients. Transplantation. (2011) 92:1237–43. doi: 10.1097/TP.0b013e31823411d7 22124283

[24] Schumacher L Leino AD Park JM. Tacrolimus intrapatient variability in solid organ transplantation: A multiorgan perspective. Pharmacotherapy. (2021) 41:103–18. doi: 10.1002/phar.v41.1 33131078

[25] Hanghoj S Boisen KA. Self-reported barriers to medication adherence among chronically ill adolescents: a systematic review. J Adolesc Health. (2014) 54:121–38. doi: 10.1016/j.jadohealth.2013.08.009 24182940

[26] Dobbels F Ruppar T De Geest S Decorte A Van Damme-Lombaerts R Fine RN. Adherence to the immunosuppressive regimen in pediatric kidney transplant recipients: a systematic review. Pediatr Transpl. (2010) 14:603–13. doi: 10.1111/j.1399-3046.2010.01299.x 20214741

[27] Pape L DeZwaan M Nöhre M Klewitz F Kyaw Tha Tun E Prüfe J. A multimodal aftercare intervention improves the outcome after kidney transplantation - results of the KTx360 degrees aftercare program using claims data. EClinicalMedicine. (2024) 73:102652. doi: 10.1016/j.eclinm.2024.102652 38841709 PMC11152610

[28] Penkower L Amanda M Ellis D Sereika SM Kitutu JMM Shapiro R. Psychological distress and adherence to the medical regimen among adolescent renal transplant recipients. Am J Transplant. (2003) 21:1418–25. doi: 10.1046/j.1600-6135.2003.00226.x 14525604

[29] Dobbels F Decorte A Roskams A Van Damme-Lombaerts R. Health-related quality of life, treatment adherence, symptom experience and depression in adolescent renal transplant patients. Pediatr Transplant. (2010) 14:216–23. doi: 10.1111/j.1399-3046.2009.01197.x 19497017

[30] Berney-martinet S Key F Bell L Lepine S Clermont M Fombonne E. Psychological profile of adolescents with a kidney transplant. Pediatr Transplant. (2009) 13:701–10.13. doi: 10.1111/j.1399-3046.2008.01053.x 18992062

[31] Creemans J Eiser C Blades M. Factors influencing agreement between child self-report and parent proxy-reports on the Pediatric Quality of Life Inventory™ 4.0 (PedsQL™) generic core scales. Health Qual Life Outcomes. (2006) 4:58. 16942613 10.1186/1477-7525-4-58 PMC1564004

[32] Mack JW McFatrich M Withycombe JS. Agreement between child self-report and caregiver-proxy report for symptoms and functioning of children undergoing cancer treatment. JAMA Pediatr. (2020) 174:e202861. doi: 10.1001/jamapediatrics.2020.2861 32832975 PMC7445628

[33] Haavisto A Korkman M Holmberg C Jalanko H Qvist E. Neuropsychological profile of children with kidney transplants. Nephrol Dial Transpl. (2012) 27:2594–601. doi: 10.1093/ndt/gfr650 22140125

[34] Gulleroglu K Baskin E Bayrakci US Aydogan M Alehan F Kantar A. Neurocognitive functions in pediatric renal transplant patients. Transplant Proc. (2013) 45:3511–3. doi: 10.1016/j.transproceed.2013.08.105 24314945

[35] Khanna D Khadka J Mpundu-Kaambwa C Lay K Russo R Ratcliffe J. Are we agreed? Self- versus proxy-reporting of pediatric health-related quality of life (HRQoL) using generic preference-based measures: A systematic review and meta-analysis. PharmacoEconomics. (2022) 40:1043–67. doi: 10.1007/s40273-022-01177-z PMC9550745 35997957

